# Immune Cell Responses and Cytokine Profile in Intestines of Mice Infected with *Trichinella spiralis*

**DOI:** 10.3389/fmicb.2017.02069

**Published:** 2017-10-31

**Authors:** Jing Ding, Xue Bai, Xuelin Wang, Haining Shi, Xuepeng Cai, Xuenong Luo, Mingyuan Liu, Xiaolei Liu

**Affiliations:** ^1^Key Laboratory of Zoonosis Research, Ministry of Education, Institute of Zoonosis/College of Veterinary Medicine, Jilin University, Changchun, China; ^2^Mucosal Immunology Laboratory, Pediatric Gastroenterology Unit, Massachusetts General Hospital East, Boston, MA, United States; ^3^China Institute of Veterinary Drugs Control, Beijing, China; ^4^State Key Laboratory of Veterinary Etiological Biology, Key Laboratory of Veterinary Parasitology of Gansu Province, Lanzhou Veterinary Research Institute, CAAS, Lanzhou, China; ^5^Jiangsu Co-innovation Center for Prevention and Control of Important Animal Infectious Diseases and Zoonoses, Yangzhou, China

**Keywords:** *Trichinella spiralis*, intestinal phase, eosinophils, goblet cells, mucosal mast cells, 33D1+ DCs, Th1/Th2

## Abstract

The intestinal phase is critical for trichinellosis caused by *Trichinella spiralis* (*T. spiralis*), as it determines both process and consequences of the disease. Several previous studies have reported that *T. spiralis* induces the initial predominance of a Th1 response during the intestine stage and a subsequent predominance of a Th2 response during the muscle stage. In the present study, immune cells and cytokine profile were investigated in the intestine of mice infected with *T. spiralis*. The results showed that the number of eosinophils, goblet cells, mucosal mast cells, and 33D1+ dendritic cells (DCs) increased during the intestinal phase of the infection. Among these, eosinophils, goblet cells, and mucosal mast cells continued to increase until 17 days post infection (dpi), and the number of 33D1+ DCs increased compared to wild type; however, it did not change with the days of infection. The mRNA and protein levels of Th1 cytokines IL-2, IL-12, and IFN-γ and the Th2 cytokines IL-4, IL-5, IL-10, IL-13, and TGF-β were all increased in the tissues of the small intestine in infected mice; however, in general, Th2 cytokines increased more than Th1 cytokines. In conclusion, our findings suggest that *T. spiralis* infection can induce an increase of small intestine mucosal immune cells and add further evidence to show that the intestinal mucosal immune system of infected mice was induced toward mixed Th1/Th2 phenotypes with the predominance of Th2 response at the early stage of infection.

## Introduction

Infection with the worm *Trichinella spiralis* (*T. spiralis*) causes Trichinellosis, which is a parasitic disease caused by eating raw or undercooked pork or the meat of wild mammals that have been infected with *T. spiralis*. Pathological processes of trichinellosis have been divided in intestinal phase and muscle phase (Chen et al., 2013). The muscle larvae (ML) of *T. spiralis* invade the intestine, settle into epithelial cells, where they grow to adulthood, mate, and procreate newborn larvae (NBL) during 3–7 days post infection (dpi). Adult *T. spiralis* are then expelled from the intestine within 10–17 dpi according to our previous results (Ding et al., 2016); therefore, we considered the first 17 dpi as intestinal phase. The intestinal phase is a critical stage of trichinellosis, since it determines both the process and consequence of the disease (Piekarska et al., 2011). The presence of worms in the intestine has been shown to induce a series of pathological changes, resulting in acute inflammatory responses within the small intestine (Khan and Collins, 2004).

This early intestinal immune response is a result of the interaction of cytokines and intestinal mucosal immune cells such as DCs, eosinophils, goblet cells, and mast cells. Evidence shows that the changes in cells described above are related to the cytokines produced by Th2 cells. Dendritic cells (DCs) are likely playing a key role in the regulation of the mucosal immune response as one of the most important antigen presenting cells in the intestine. DCs are central to the polarization of CD4+ T cells, and several studies have demonstrated that the excretion/secretion (ES) product of *T. spiralis* or the infection itself can induce a strong Th2 response. IL-5-dependent eosinophils have been shown to contribute to worm expulsion in *T. spiralis* infection (Vallance et al., 1999). Ishikawa et al. (1997) suggested that small intestinal goblet cell hyperplasia is probably regulated by Th2 cells in mice that have been infected with *T. spiralis*, while Th2-derived cytokines (different from IL-5) induce stem cells to preferentially differentiate along the goblet cell lineage. The intestinal mastocytosis depends on the interplay between the stem cell factor (SCF) and cytokines produced mainly by CD4+ Th2 cells (such as IL-3, IL-4, and IL-9), contributing to the host defense against trichinella infection (Abe et al., 1988; Madden et al., 1991; Faulkner et al., 1997).

However, several other previous studies have suggested that the immune system was induced in response to the initial predominance of a Th1 response during the intestinal stage and subsequent predominance of a Th2 response during the muscle stage of *T. spiralis* infection. The mRNA expressions of IFN-γ and IL-12 were upregulated at the early stage of infection, and the expression of IL-4, IL-10, and IL-13 increased during the muscle stage (Yu et al., 2013a). A further report showed that during the early stage of the intestinal phase of *T. spiralis* infection, the host triggers a Th1-type immune response with aimed at eliminating the parasite (Munoz-Carrillo et al., 2017).

The two conclusions above are contradictory; therefore, the aim of our study was to investigate the populations of eosinophils, goblet cells, mast cells, 33D1+ DCs, and the production of Th1 and Th2 cytokines in mice infected with *T. spiralis*, to deepen our understanding of the interplay between mucosal immune cells and cytokines, while identifying Th cell phenotypes during the early stage of infection.

## Materials and Methods

### Animals and Parasite Infections

*Trichinella spiralis* (ISS534) were maintained in ICR mice via serial passages of the infection into new hosts. ML were recovered from infected mice via artificial digestion (magnetic stirrer method) according to OIE standard protocol (OIE, 2016). Female ICR mice, aged 6–8 weeks, were obtained from the Norman Bethune University of Medical Science (NBUMS), China. A total of 35 female ICR mice were randomly divided into seven groups and the infected groups were infected with 300 ML each mouse.

Animals were treated according to the guidelines of the National Institute of Health (publication No. 85–23, revised 1996). Animal protocols have been reviewed and approved by the Ethical Committee of the Jilin University affiliated with the Provincial Animal Health Committee, Jilin Province, China (Ethical Clearance number IZ-2009-08).

### Histology (Tissue Embedding and Slicing)

A length of about 1 cm of duodenum in the small intestine invaded by T. spiralis (see **Supplementary Figure S1**) was taken from mice, then fixed with formalin, embedded in paraffin, and sliced with a microtome. The thickness of each slice was approximately 5 μm. Some of these tissue slices were stained with hematoxylin and eosin (H&E) for histological examinations of eosinophils, while the remaining tissue slices were used for immunohistochemical staining to evaluate goblet and mast cells. Three to five Peyer’s patches (PPs) were dissected from mice intestines, embedded, and sliced with the same methods described above to evaluate DCs.

### Eosinophils Identification

The slices were deparaffinized, rehydrated, and stained with H&E. The granules in eosinophils were dyed red and positive cells were observed via light microscopy at a magnification of ×200 (at least three sections were examined per animal).

### Goblet and Mast Cell Counts

Small intestine tissue sections were deparaffinized and subsequently treated in sodium citrate buffer (pH 6.0) with a 500-watt microwave oven for 12 min to improve antigen retrieval. Then, tissue slices were treated with 3% hydrogen peroxide solution to block activity of endogenous peroxidase. Non-specific binding was blocked via incubation in PBS, containing 5% normal goat serum for 30 min at room temperature. For goblet cells, sections were incubated with anti-MUC2 rabbit polyclonal antibody (1:400 dilutions; Abcom, United States) overnight at 4°C. For mast cells, sections were incubated with anti-mast cell tryptase antibody (AA1) (1:200 dilutions; Abcom, United States) overnight at 4°C. Then, the slices were washed with PBS, and incubated with peroxidase-conjugated anti-rabbit secondary antibody (Beijing Biosynthesis Biotechnology Co. Ltd., China) for 30 min at room temperature, followed by 3, 3′-diaminobenzidine (DAB) and hematoxylin staining respectively. Images were captured using an Olympus BX53 fluorescence microscope (Tokyo, Japan). The number of cells that were stained with the primary antibody was counted in all the photographs by three researchers who were blinded to the animal groupings, and the cell numbers were expressed as visible goblet cells or mast cells per field.

### Immunofluorescence of DCs in PPs

Peyer’s patches tissue sections (5 μm) were blocked in 5% bovine serum albumin (BSA) and then incubated with DC marker (33D1) (200 μg/ml; Santa Cruz) overnight at 4°C. Slides were washed thrice in PBS for 15 min. Sections were then incubated with goat polyclonal Cy3-conjugated secondary antibodies (705-165-147; Jackson ImmunoResearch) against mouse immunoglobulin (0.5 μg/ml; F6257; Sigma-Aldrich) and multiple sites were examined for each of the PPs at ×200 magnification via laser scanning confocal microscopy.

### Quantitative Real-time Polymerase Chain Reaction for mRNA Expression

Approximately 100 mg small intestine tissues per mouse were homogenized in TRIzol reagent, and total RNA was extracted via the RNA Extraction Kit (TaKaRa). Total RNA purity was measured with a spectrophotometer. RNA was treated with DNase-1 prior to reverse transcription. To determine the mRNA levels of Th1 cytokines inter-leukin (IL)-2, IL-12, and Interferon (IFN)-γ and the Th2 cytokines IL-4, IL-5, IL-10, IL-13, and the transforming growth factor-β (TGF-β) in the tissues of the small intestine, real-time quantitative PCR was conducted using Power Sybr^®^Green (Applied Biosciences) on a Bio-Rad CFX 96 Real Time System C100 Thermal Cycler. Relative gene expression levels were determined via normalization to GAPDH level, using the ΔΔCt method. All primers are listed in **Table 1**.

**Table 1:** Primer sequences used for qRT-PCR to identify the mRNA transcript.

Genes	Primer sequence (5′-3′)		Accession no.
IL-2	Forward primer Reverse primer	ATGTACAGCATGCAGCTCGCATCCTGTGTCA AGTCAAATCCAGAACATGCCGCAGACGTCCA	NM_008366.3
IL-12α	Forward primer Reverse primer	CAGAAAGGTGCGTTCCTCGT GGAACACATGCCCACTTGCT	NM_008351.3
IFN-γ	Forward primer Reverse primer	CCATCGGCTGACCTAGAGAA GATGCAGTGTGTAGCGTTCA	NM_008337.4
IL-4	Forward primer Reverse primer	ACAGGAGAAGGGACGCCAT GAAGCCCTACAGACGAGCTCA	NM_021283.2
IL-5	Forward primer Reverse primer	TCAGCTGTGTCTGGGCCACT TTATGAGTAGGGACAGGAAGCCTCA	NM_010558.1
IL-10	Forward primer Reverse primer	AGCCGGGAAGACAATAACTG CATTTCCGATAAGGCTTGG	NM_010548.2
IL-13	Forward primer Reverse primer	TCTTGCTTGCCTTGGTGGTC GGTCTTGTGTGATGTTGCTCAGC	NM_008355.3
TGF-β	Forward primer Reverse primer	GCTGTGAAGCCTTGAGAGTAATGG TTCCTGTTGACTGAGTTGCGATAA	NM_011577.2
β-Actin	Forward primer Reverse primer	TGGAATCCTGTGGCATCCATGAAAC TAAAACGCAGCTCAGTAACAGTCCG	NM_007393.5

### Measurements of Cytokines in Serum

At 0, 1, 3, 7, 11, and 17 dpi, serum was separated from blood that was extracted from the mouse eye socket. Commercial enzyme-linked immunosorbent (ELISA) sets (R&D Systems, Minneapolis, MN, United States) were used to measure the concentration of the Th1 cytokines IL-2 and IFN-γ and the Th2 cytokines IL-4 and IL-10 according to manufacturer’s instructions.

### Statistical Analysis

Data are presented as means ± standard error of the means (SEM). Differences were analyzed either via the Student’s *t*-test or one-way ANOVA. ^∗^*P*-value < 0.5, ^∗∗^*P*-value < 0.01, and ^∗∗∗^*P*-value < 0.001 were regarded as statistically significant.

## Results

### Eosinophils in the Small Intestines

**FIGURE 1:**
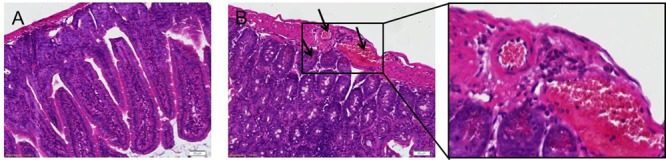
H&E staining of eosinophils. Representative inverted microphotographs showing eosinophils (red-stained cells) within the duodenum of control **(A)** and infected mice at 7 dpi **(B)**.

Eosinophils infiltration was apparent in mice infected with *T. spiralis* (**Figure 1B**) during the intestinal phase compared to the control group (**Figure 1A**, observed via light microscopy). As shown in **Figure 1B**, the eosinophils increased significantly in the intestinal at 7 dpi, as well as for the other days of infection (data not shown).

### Goblet Cells Count

**FIGURE 2:**
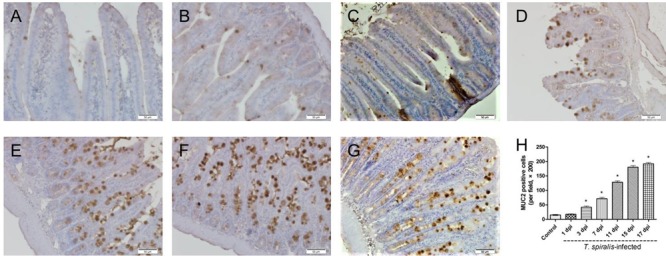
Immunohistochemistry of goblet cells. **(A–G)** Representative microphotographs showing MUC2-immunopositive cells (corresponding to goblet cells) in the small intestine of control **(A)** and previously infected mice at 1 dpi **(B)**, 3 dpi **(C)**, 7 dpi **(D)**, 11 dpi **(E)**, 15 dpi **(F)**, and 17 dpi **(G)**; scale bar: 50 μm. **(H)** Quantification of goblet cells (number per field, ×200). Data show mean ± SEM of three animals per group. ^∗^*p* < 0.05 vs. control group.

Goblet cells appeared dark brown in color after immunohistochemical staining. In the control group, only few goblet cells could be seen in the small intestine (**Figure 2A**), while significantly higher numbers of goblet cells were observed in the villi of mice infected with *T. spiralis* (**Figures 2B–G**). As shown in **Figure 2H**, the number of goblet cells increased up until 17 dpi in the intestinal phase compared to uninfected mice (*P* < 0.01).

### Mucosal Mast Cells Count

**FIGURE 3:**
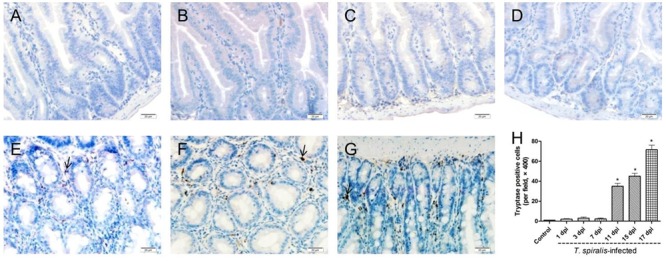
Immunohistochemistry of colonic mucosal mast cells. **(A–G)** Representative microphotographs showing tryptase-immunopositive cells (corresponding to mucosal mast cells, MMCs) in the colonic mucosa of control **(A)** and previously infected mice at 1 dpi **(B)**, 3 dpi **(C)**, 7 dpi **(D)**, 11 dpi **(E)**, 15 dpi **(F)**, and 17 dpi **(G)**; scale bar: 20 μm. **(H)** Quantification of colonic MMCs (number per field, ×200). Data show mean ± SEM of three animals per group. ^∗^*p* < 0.05 vs. control group.

Mast cells appeared dark brown after immunohistochemical staining for tryptase. As shown in **Figure 3**, few mast cells were observed in the duodenum of control mice, while numerous positively stained mast cells could be seen in the small intestine tissues of *T. spiralis* infected mice, reaching the highest number at 17 dpi (*P* < 0.01).

### DCs Counts

**FIGURE 4:**
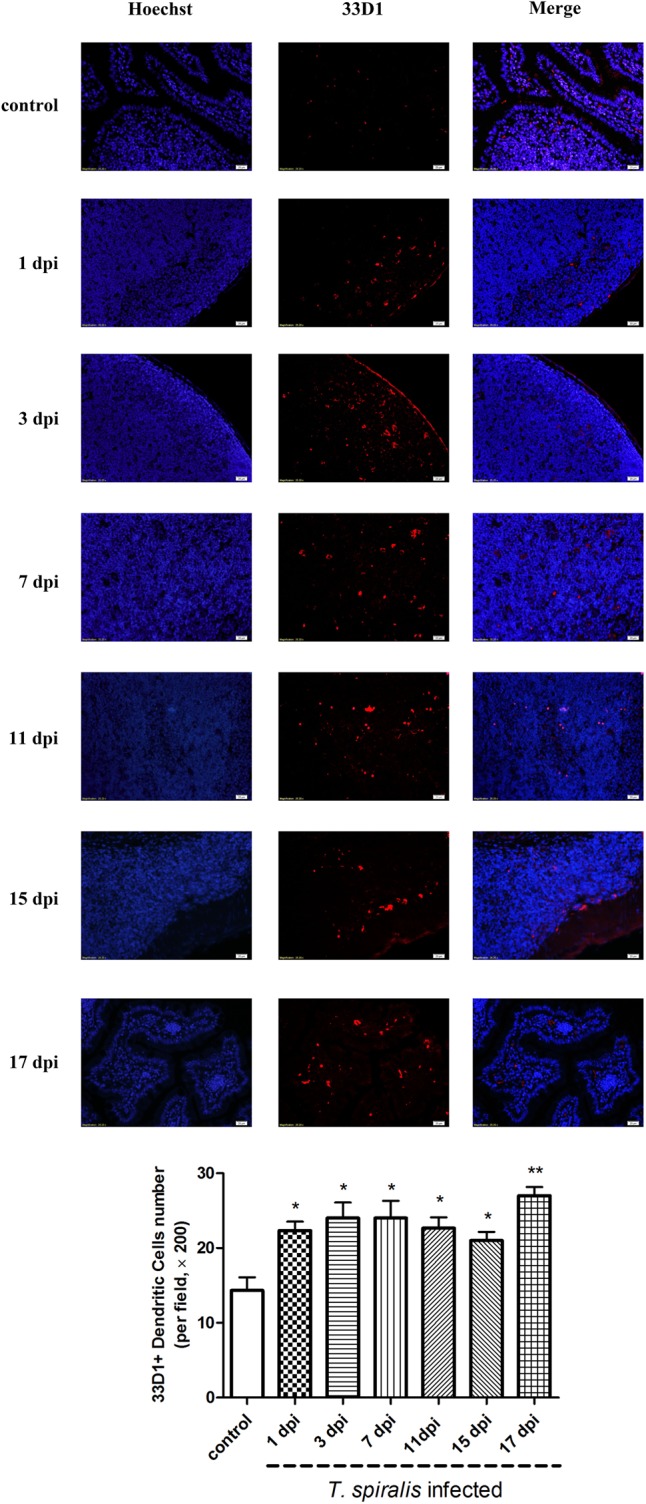
Immunofluorescence of dendritic cells (DCs). Representative confocal microphotographs showing 33D1-immunopositive cells (corresponding to DCs, red-stained cells) in the Peyer’s Patch of control and previously infected mice at 1, 3, 7, 11, 15, and 17 dpi; scale bar: 20 μm. And quantification of 33D1+ DCs (number per field, ×200). Data show mean ± SEM of three animals per group. ^∗^*p* < 0.05 vs. control group.

Dendritic cells play an important role in keeping the balance between Th1 and Th2 responses. In mice, this Th1/Th2 balance appears to be primarily regulated by two distinct DC subsets: CD205+ DCs (which induce Th1 polarization) and 33D1+ DCs (which elicit Th2 dominance). As shown in **Figure 4**, 33D1+ DCs were identified via immunofluorescence. According to the results, the number of 33D1+ DCs in the mice infected with *T. spiralis* increased compared to wild-type mice (*P* < 0.5); however, it did not change with the days of infection. We furthermore detected CD205+ DCs with the same method; however, the number of CD205+ DCs did not show any difference to the control group (data not shown).

### Th1/Th2 Cytokine mRNA Expression Measurement via qRT-PCR

**FIGURE 5:**
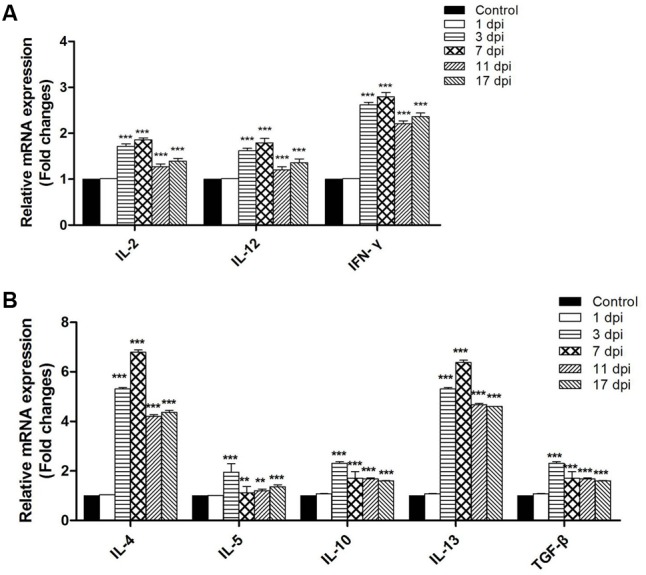
Th1 cytokines inter-leukin (IL)-2, IL-12, and Interferon (IFN)-γ **(A)** and Th2 cytokines IL-4, IL-5, IL-10, IL-13, and transforming growth factor-β (TGF-β) **(B)** mRNA levels in the tissues of the small intestine. Total RNA was purified from the small intestine and cytokine mRNAs were assayed via real-time PCR. *N* = 3 independent experiments. ^∗∗^*P* < 0.05 and ^∗∗∗^*P* < 0.01 vs. control group.

To determine whether *T. spiralis* infection altered the Th1/Th2 balance, several key cytokines of Th1 and Th2 were measured via qRT-PCR. The relative mRNA expression levels of the Th1 cytokines IL-2, IL-12, and IFN-γ as well as the Th2 cytokines IL-4, IL-5, IL-10, IL-13, and TGF-β were all increased in the intestine of mice infected with *T. spiralis* at 3, 7, 11, 15, and 17 dpi (*P* < 0.01) compared to the control group. Here, it is worth noting that Th2 cytokines increased more than Th1; therefore, we suggest that the immune system in infected mice developed toward a mixed Th1/Th2 phenotype with an emerging predominance of Th2 response (**Figure 5**).

### Th1/Th2 Cytokine Expression in Serum Measured via ELISA

**FIGURE 6:**
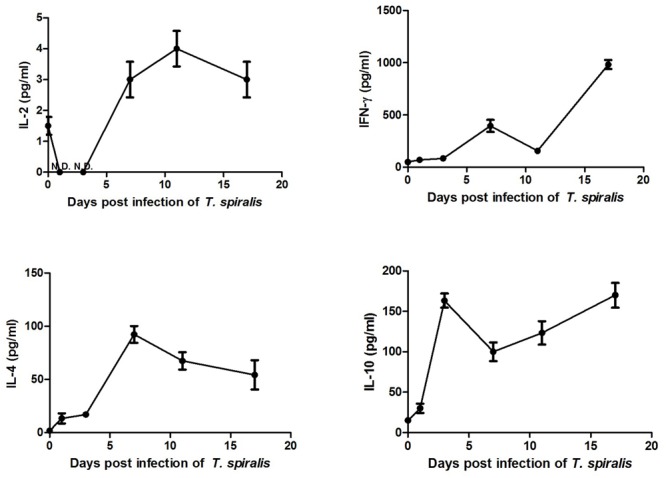
Expression of Th1 cytokines IL-2 and Interferon (IFN)-γ as well as Th2 cytokines IL-4 and IL-10 in serum. Serum was separated from blood obtained from the mouse eye socket and cytokines were detected via ELISA. Data are means ± SEM of three animals per group.

To investigate the systemic immune response of infected mice, we analyzed the serum levels of Th1/Th2 cytokines at different dpi. The expression of the Th1 cytokine IFN-γ increased marginally during the early days of infection, and increased significantly from 7 dpi onward (**Figure 6**). Expression of the Th1 cytokine IL-2 was significantly downregulated at the first 3 days of infection, when it could not be detected at all. However, it increased from 7 to 17 dpi, differing from the uninfected control. Expression of the Th2 cytokine IL-4 was upregulated during the entire intestinal phase. Th2 cytokine IL-10 was significantly upregulated during the intestinal phase. These results confirmed that infection with *T. spiralis* in mice was characterized by a mixed systemic Th1/Th2 with predominance of the Th2 response during the intestinal phase.

## Discussion

Here, we investigated the intestinal mucosa immune conditions during the intestinal stage of *T. spiralis* infection. The results showed time-associated changes within intestinal immune cell populations and Th1/Th2-related cytokines in *T. spiralis* infected mice during the intestinal phase. The number of eosinophils, goblet cells, and mast cells gradually increased during the entire intestinal phase and the highest numbers were reached on 17 dpi. The number of 33D1+ DCs increased compared to the control group; however, it did not change significantly with the days of infection during this period. According to the cytokine expression in both mRNA and protein level, we found that *T. spiralis* can induce a complex Th1/Th2 response in the immune system with predominant polarization to Th2 during the early stage of infection. During the chronic phase of human trichinellosis, it was also a mixed Th1/Th2 response and the Th2 response play a predominant role in the cellular immune response (Della Bella et al., 2017).

Eosinophilia is a characteristic of the host immune response to *T. spiralis*. Infection with *T. spiralis* has been reported to stimulate an increase of eosinophils (Herndon and Kayes, 1992) and eosinophils have been reported to be able to resist parasite infection, including *T. spiralis* (Klion and Nutman, 2004). According to Chen et al. (2013) the number of eosinophils was elevated in mice infected with *T. spiralis* at 10 dpi. In this study, we observed a significantly increased number of eosinophils in mice infected with *T. spiralis* during the intestinal phase. Eosinophils can kill new-born larvae (NBL) of *T. spiralis* (Kazura and Aikawa, 1980) and Th2 cytokine IL-5 can stimulate the cytotoxicity of eosinophils *in vitro* (Lee, 1991). Moreover, IL-5-dependent eosinophils contribute to worm expulsion during *T. spiralis* infection (Vallance et al., 1999). However, one recent study suggested that eosinophils entered the infection sites immediately after tissue invasion by *T. spiralis* larvae, which is vital for *T. spiralis* survival. Furthermore, eosinophils expand DCs and CD4+ T cells by producing IL-10, to inhibit the activation of macrophages and neutrophils that may threaten parasite larvae by releasing NO (Huang et al., 2014). Thus, it still remains a question whether eosinophils actually contribute to the host defense against *T. spiralis* or benefit parasite growth and survival.

During the intestinal phase, goblet cell hyperplasia is also an important feature of intestinal nematode infection (Marshman et al., 2002). The number of goblet cells increased on 7, 10, and 15 dpi in *T. spiralis* infected mice (Yu et al., 2013b). Similar results have been obtained in mice infected with *T. spiralis* at 10 dpi (Chen et al., 2013). Coincidentally, the goblet cell numbers in *T. spiralis* infected mice increased both at 7 and 10 dpi compared to control group, and this number was higher at 10 dpi than at 7 dpi (Piekarska et al., 2011). All these results are similar to those found in our study. Goblet cells secrete types of active molecules, among which mucus is known to create a physical barrier on mucosal surfaces of the small intestine. A number of studies have suggested that Muc-2 (secreted) and Muc-3 (membrane bound) are both upregulated in the small intestine of mice infected with *T. spiralis*; however, relatively little data exists about their function. With the use of numerous experimental models, Th2 cytokines have been shown to be essential for goblet cell hyperplasia, especially IL-4 and IL-13 (Fallon et al., 2002; Kuperman et al., 2005). Activation of the IL-4/IL-13 receptor IL-4Ra and the activation of downstream STAT6 are also essential for goblet cell hyperplasia. Goblet cell hyperplasia has not been observed in Stat6 or IL-4Ra deficient mice infected with *T. spiralis*, so that worms cannot be expelled (Khan et al., 2001; Horsnell et al., 2007). Thus, goblet cell hyperplasia observed in *T. spiralis* infections has been suggested to be a mucosal epithelial response to Th2 cytokines, which is mainly controlled by IL-13 (Knight et al., 2008). However, the increased Th1 cytokine IFN-γ in the dry eye disease has been shown to induce a lack of goblet cells and mucin deficiency. This could also be typically found in respiratory and gastrointestinal tracts of hosts and IFN-γ could function similarly both in rats and humans (Garcia-Posadas et al., 2016). According to our results, the Th2 cytokine IL-13 and Th1 cytokine IFN-γ both increased; therefore, the changes of goblet cell numbers are regulated by both Th1 and Th2 cytokines, which is a dynamic balancing process in infected mice.

*Trichinella spiralis* infected mice showed mastocytosis in the intestinal mucosa on 10 and 15 dpi (Yu et al., 2013b). It has been reported that only few mast cells exist in the small intestine of wild-type mice, while more mast cells have been observed in mice infected with *T. spiralis* (Chen et al., 2013). Here, we found that the number of mast cells did not change significantly during the early days after infection, but started to increase from 11 dpi. Mast cell hyperplasia has been reported to temporally occur alongside worm expulsion (Pennock and Grencis, 2006). Proteases secreted by mast cells, including the mouse mast cell protease 1 (Mcpt-1), have the ability to expel worms from the small intestine. A delay in worm expulsion was observed in mast cell-deficient mice (WW^v^) (Tuohy et al., 1990; Miller, 1996) and Mcpt-1-null mice (Knight et al., 2000), and mast cell reduction in infected mice also significantly induced worm expulsion delay (Alizadeh and Murrell, 1984). Intestinal mastocytosis is dependent on the interplay between the SCF and cytokines produced mainly by CD4+ Th2 cells, such as IL-3, IL-4, and IL-9, contributing to host defense against *T. spiralis* infection (Abe et al., 1988; Madden et al., 1991; Faulkner et al., 1997). Mcpt-2 was also increased in intestinal epithelial cells (IECs) invaded by *T. spiralis* (Ming et al., 2016); however, the role of Mcpt-2 remains speculative and a subject for future investigation. In addition, studies have shown that mast cells contribute to type 2 cytokine–mediated inflammation, which is necessary for the development of protective immunity to helminth parasites such as *T. spiralis*; however, the inhibition of mast cell responses has been associated with reduced inflammation and a loss of protective immunity (Henry et al., 2016). Interestingly, a report has shown that mast cell-triggered DC modulation promotes the induction of Th1 and Th17 responses (Dudeck et al., 2011). Therefore, the mastocytosis induced by *T. spiralis* depends on a complicated interplay between Th1 and Th2 responses, similar to that of goblet cells described above.

Dendritic cells play an important role in retaining the immune balance between Th1 and Th2 responses (Neubert et al., 2014). In mice, this Th1/Th2 balance appears to be regulated by two distinct DC subsets (Neubert et al., 2014): CD205+ DCs, which induce Th1 polarization, and 33D1+ (Nussenzweig et al., 1982) DCs, which elicit Th2 dominance. In the present study, the number of 33D1+ DCs increased slightly in the PPs of mice infected with *T. spiralis* compared to control mice, while the number of CD205+ DCs showed no difference in comparison to the control group. Although *T. spiralis* ES L1 antigens did not affect the expression pattern of surface markers (i.e., phenotype of DC), it did affect the cytokine release from DCs. Previous studies showed that *T. spiralis* ES-stimulated DCs increased the production of IL-4, IL-10, and TGF-β, while decreased production of IFN-γ and IL-17 (Sofronic-Milosavljevic et al., 2013), resulting in a Th2 response (Cvetkovic et al., 2014). According to our results for the Th1/Th2 cytokines expression at mRNA level, the production of IL-4, IL-10, and TGF-β increased more than 3-fold compared to the control group, while IFN-γ increased only about 2.5-fold. In serum, IFN-γ increased slightly and IL-2 was downregulated during early days of infection, while Th2 cytokines IL-4 and IL-10 increased during the whole intestinal phase. In summary, these observations demonstrate that *T. spiralis* infection caused DCs to gain immunomodulatory capacities and induced them to drive a predominant Th2-polarized response. 33D1+ DCs not only induced Th0 cells to Th2 cells, but also influenced the frequency and function of Treg cells. The frequency and function of IL-2–dependent Treg cells depends on the presentation of MHC II–restricted autoantigens to self-reactive CD4+ T cells via 33D1+ DCs (Stolley and Campbell, 2016).

The *T. spiralis* induced immune response is a complicated process both in intestinal mucosal immune responses and systemic immune responses. Our previous results have suggested that systemic immune responses have been suppressed during the early stage of infection and that macrophages were induced to M2 (Ding et al., 2016). *T. spiralis* infection caused a series of pathological changes within the infected intestine, which contained intestinal eosinophilia infiltration, goblet cell hyperplasia, and mucosal mast cell hyperplasia. All these cells that collaborated with DCs and Th1/Th2 cytokines influenced the initiation stage of the anti-parasite response and expelled worms from the small intestine.

## Conclusion

We found that *T. spiralis* infection induced a mixed Th1/Th2 response with a predominant Th2 response during the early phase of infection. This context provides new evidence that *T. spiralis* can change the immune balance of the host, enabling a better understanding of the immune status during the early stage of infection. Given the increasing awareness of the role of *T. spiralis* for triggering and regulating immune responses, more effort is required to search for effective immune modulators in *T. spiralis* excretory-secretory products to prevent or/and treat autoimmune diseases such as multiple sclerosis (MS), type 1 diabetes mellitus (T1DM), and inflammatory bowel disease (IBD).

## Author Contributions

XW, XC, and XuL designed the study; JD and XB conducted the experiments. HS, ML, and XiL planned and supervised the experiments, and JD contributed to the writing of the manuscript.

## Conflict of Interest Statement

The authors declare that the research was conducted in the absence of any commercial or financial relationships that could be construed as a potential conflict of interest.

## Supplementary Material

FIGURE S1 Invasion of Trichinella spiralis into small intestine.
