# Supplementary figures and images for: Immune Cell Responses and Cytokine Profile in Intestines of Mice Infected with Trichinella spiralis
Source: Front Microbiol. 2017 Oct 31;8:2069. doi: 10.3389/fmicb.2017.02069 (PMC5671581; doi:10.3389/fmicb.2017.02069)

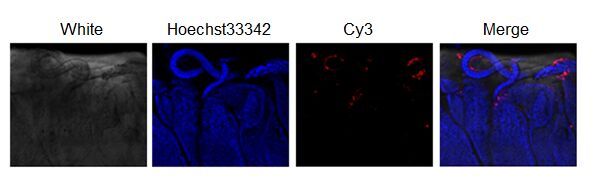

Supplement: FIGURE S1 — Invasion of Trichinella spiralis into small intestine. [file Image_1.JPEG]
